# Ectopic or Over-Expression of Class 1 Phytoglobin Genes Confers Flooding Tolerance to the Root Nodules of *Lotus japonicus* by Scavenging Nitric Oxide

**DOI:** 10.3390/antiox8070206

**Published:** 2019-07-04

**Authors:** Mitsutaka Fukudome, Eri Watanabe, Ken-ichi Osuki, Nahoko Uchi, Toshiki Uchiumi

**Affiliations:** Graduate School of Science and Engineering, Kagoshima University, 1-21-35 Korimoto, Kagoshima 890-0065, Japan

**Keywords:** flooding, hemoglobin, hypoxia, *Lotus japonicus*, *Mesorhizobium loti*, nitric oxide, nitrogen fixation, ROS, symbiosis

## Abstract

Flooding limits biomass production in agriculture. Leguminous plants, important agricultural crops, use atmospheric dinitrogen gas as nitrogen nutrition by symbiotic nitrogen fixation with rhizobia, but this root-nodule symbiosis is sometimes broken down by flooding of the root system. In this study, we analyzed the effect of flooding on the symbiotic system of transgenic *Lotus japonicus* lines which overexpressed class 1 phytoglobin (*Glb1*) of *L. japonicus* (*LjGlb1-1*) or ectopically expressed that of *Alnus firma* (*AfGlb1*). In the roots of wild-type plants, flooding increased nitric oxide (NO) level and expression of senescence-related genes and decreased nitrogenase activity; in the roots of transgenic lines, these effects were absent or less pronounced. The decrease of chlorophyll content in leaves and the increase of reactive oxygen species (ROS) in roots and leaves caused by flooding were also suppressed in these lines. These results suggest that increased levels of Glb1 help maintain nodule symbiosis under flooding by scavenging NO and controlling ROS.

## 1. Introduction

Flooding often reduces crop growth and yield, causing serious problems for farmers. Plant growth is hampered by flooding because it exposes plants to hypoxia, which inhibits aerobic respiration and photosynthesis, reducing ATP production. Hypoxia inhibits photosynthesis by inducing production of reactive oxygen species (ROS), which damage the chloroplast membrane and decrease the photosynthetic potential [1,2]. Excess ROS also lead to lipid peroxidation and alterations in lipid composition, electrolyte leakage, and ultimately cell death [3,4,5]. Another reactive molecule, nitric oxide (NO), is produced in plants in response to biotic and abiotic stresses, including hypoxia [6,7,8,9]. NO serves as a signal molecule in various physiological and pathogenic responses of plants such as stomatal opening and closing [10], protein *S*-nitrosylation, and cGMP nitration [11]. Excess NO is toxic and inhibits plant growth; plants regulate NO levels by producing plant hemoglobin (phytoglobin, Glb) [12,13].

Glbs are divided into three classes: Glb1, Glb2, and Glb3 [14,15,16,17]. Leghemoglobin (Lb) of leguminous plants, which was the first identified Glb [18], belongs to Glb2 and is essential for legume–rhizobia symbiosis because it regulates oxygen partial pressure in root nodules [19]. Although the function of Glb3 is unknown, it may interact with NO [20]. Glb1 has extremely high affinity for oxygen [21] and scavenges NO by oxidizing it to nitrate [12,13,17,22]. Under hypoxia, overexpression of Glb1 ameliorates the energy status and growth of both maize cells and alfalfa roots [6,8,12], and enhances the survival of *Arabidopsis thaliana* [16]; in all cases, consistent low NO level strongly suggests the role of NO-scavenging activity of Glb1 in tolerance to hypoxia. Overexpression or ectopic expression of tobacco gene *NtHb1* enhances Cd tolerance by reducing Cd and NO levels in *Nicotiana tabacum* and *A. thaliana* [23,24]. At least eight *Glb* genes have been identified in the genome of *Lotus japonicus*: two *Glb1*s (*LjGlb1-1*, *LjGlb1-2*), four *Glb2*s (*LjGlb2* and three Lb genes) and two *Glb3*s (*LjGlb3-1*, *LjGlb3-2*) [25,26]. *LjGlb1-1* is the only NO-inducible *Glb* gene of *L. japonicus* [26].

The NO-scavenging activity of Glb1 is required for establishing proper root nodule symbiosis [27]. In the *L. japonicus–Mesorhizobium loti* symbiosis, inoculation with *M. loti* induces NO production in roots with the simultaneous expression of the *Glb1* gene (*LjGlb1-1*) [28]. NO inhibits nitrogenase [29,30] and promotes nodule senescence [31]. A null mutant line of *LjGlb1-1* shows low infection and low nitrogenase activity of the nodules [27], whereas overexpression of *LjGlb1-1* increases nitrogenase activity [32,33]. The beneficial effects of *LjGlb1-1* overexpression may be attributed to a reduced level of NO [33]. No drastic differences have been observed in the shape and growth of plants among these overexpression lines, the null mutant, and the wild type with supply of nitrogen source [27,33], although the timing of bolting and flowering has not been statistically compared. In the *Alnus firma* (actinorhizal plant)–*Frankia* (actinobacterium) symbiosis, Glb1 of *A. firma* (AfGlb1, accession number AB221344 in DDBJ database) may support the nitrogenase activity of the nodules as a NO scavenger [34].

Flooding adversely affects nodule symbiosis; NO produced in the nodules in response to flooding decreases nitrogenase expression and activity [35,36,37]. NO might attack nodule cells during flooding and delay the recovery of the symbiotic activity of the nodules after flooding. Because NO-scavenging activity contributes to hypoxia tolerance, we hypothesized that *Glb1* overexpression might improve the tolerance of nodule symbiosis to hypoxia.

In this study, we examined the tolerance of the nodule symbiosis to flooding in two transgenic *L. japonicus* lines that express *LjGlb1-1* or *AfGlb1* driven by the CaMV 35S promoter. Our results suggest that Glb1 overexpression improves nodule symbiosis by controlling not only NO but also ROS.

## 2. Materials and Methods

### 2.1. Biological Materials

*Lotus japonicus* accession Gifu B-129 and its derivative lines were used as host plants. The null mutant line 30096642 (abbreviated hereafter as 96642), bearing the *LORE1* retrotransposon inserted in the 5′-untranslated region of *LjGlb1-1* [27], was obtained from the *LORE1* collection [38,39,40]. Binary vectors carrying the constitutive cauliflower mosaic virus 35S (CaMV 35S) promoter and cDNA of *LjGlb1-1* or *AfGlb1* were constructed with pIG121-Hm, and the lines of *L. japonicus* expressing these constructs (referred to as Ox1 [33] and as Afx1, respectively) were produced according to Aoki et al. [41]. *Mesorhizobium loti* MAFF303099 [42] was used as a microsymbiont of *L. japonicus*.

### 2.2. Growth Conditions and Flooding Treatment

*Lotus japonicus* B-129 and its derivatives were germinated and grown as described previously [28]. In brief, 5 days after germination, seedlings were transferred to pots filled with vermiculite moistened with Fåhraeus liquid medium [43] and inoculated with *M. loti* MAFF303099 suspension in water (10^7^ cells mL^−1^) [42]. The plants were grown under photosynthetically active radiation of 150 μmol photons m^−2^ s^−1^ (16-h photoperiod) at 25 °C for 5 weeks after inoculation. At 4 weeks after inoculation, the pots were put in wider containers filled with distilled water, so that the water level was maintained 1 cm above the soil surface for 1 week. Plants not subjected to flooding were used as controls.

### 2.3. Nitrogenase Activity

Nitrogenase activity of the nodules was determined as acetylene reduction activity (ARA) according to Shimoda et al. [32]. Whole plants or nodules detached from the roots were placed in glass tubes containing wet filter paper. The tubes were filled with a mixture of acetylene (C_2_H_2_) and air (1:9 v/v). After 2 h incubation at 25°C, the amount of ethylene in the gas phase was determined by gas chromatograph (GC-3A, Shimadzu, Kyoto, Japan).

### 2.4. Endogenous NO and ROS in Roots

Endogenous NO was monitored by fluorescence microscopy as described by Nagata et al. [28]. The assay used the cell-permeable DAF-FM DA probe, which is deacetylated by intracellular esterases to DAF-FM; the latter reacts with the endogenous NO oxidation product N_2_O_3_ to form a highly fluorescent triazole. The roots were soaked for 1 h in distilled water containing 20 μM DAF-FM DA (Goryo Chemical, Sapporo, Japan). The endogenous ROS were monitored as NO, except that 10 μM cell-permeable ROS probe CellROX Deep Red Reagent (Invitrogen, NY, USA) was used. The elongation zone (1–2 cm from the root tip) was examined. Confocal images were captured under an A1si-90i microscope and epifluorescence images under an Eclipse 90i microscope (both from Nikon, Tokyo, Japan). Fluorescence intensity was quantified in ImageJ software (https://imagej.nih.gov/ij/).

### 2.5. NO Released from Nodules

The NO released from nodules was assessed by using the non-cell-permeable DAF-FM probe. The nodules were detached and immediately soaked in 7 µM DAF-FM for 10 min. The relative fluorescence units (RFUs) of the DAF-FM solution were measured by fluorometer (e-Spect2, Malcom, Japan) with excitation at 495 nm and emission at 519 nm.

### 2.6. Leaf Chlorophyll Content

Five leaves per plant were collected, chlorophyll was extracted, and absorbance (*A*) at 663.8 nm and 646.8 nm was measured and quantified according to Porra et al. [44]. The amounts of chlorophyll *a* (Chl-*a*), chlorophyll *b* (Chl-*b*), and their sum (Chl-*a+b*) were calculated as follows:

Chl-*a* = 12.00 × *A*_663.8_ − 3.11 × *A*_646.8_

Chl-*b* = 20.78 × *A*_646.8_ − 4.88 × *A*_663.8_

Chl-*a+b* = 17.67 × *A*_646.8_ + 7.12 × *A*_663.8_

The results were expressed as the ratio to the unflooded control of WT.

### 2.7. Electrolyte Leakage from Leaves

Electrolyte leakage was measured according to Rolny et al. [45]. Five leaves were floated on 5 mL of deionized water with continuous shaking on a rocking shaker (SK-R1807-E, DLAB Scientific, Beijing, China). Electrolyte content in the solution was measured immediately (C0) and after 3 h (C3) of incubation at 25°C with a conductivity meter (AS650, AS One, Osaka, Japan). Total electrolyte content (TC) was determined in the same way after incubation at 80 °C for 3 h. Electrolyte leakage rate was calculated as (C3 − C0)/TC and expressed as the ratio to untreated WT.

### 2.8. qRT-PCR Analysis of Senescence-Related Genes

Total RNA was extracted from nodules (max. 50 mg) with the RNeasy Plant Mini kit (Qiagen, Hilden, Germany). qRT-PCR was performed in a 7300 Real-Time PCR system (Applied Biosystems, Foster City, CA, USA) with a One Step SYBR Prime Script RT-PCR kit (Takara, Shiga, Japan). The reverse transcription step was 5 min at 42°C. Primers for *LjGlb1-1* (Lj3g3v3338170, 5′-CCTTTGGAGGAGAACCCCAA-3′ and 5′-GAGCTGCTGATTCACAAGTCA-3′), *heat shock protein* (Lj4g3v0473190; 5′-CAGTGGGAAATTCCAGAGGA-3′ and 5′-AGTGAGAACCCCATTCTCCA-3′), *osmotin precursor* (Lj2g3v2017460; 5′-GGACAGGTGCCATGATTCTT-3′ and 5′-GAAAGTGCTGGTGGGATCAT-3′), *cysteine protease* LjCyp2 (Lj1g3v4047250; 5′-GGAGAACAATGGGGTGAAGA-3′ and 5′-GCCACACAAACCCAATACTG-3′), and *LjeIF-4A* (Lj6g3v1382260; 5′-TGGAAGCTTCGAAGAGATGG-3′ and 5′-GTGCCAGATTGAGCCTGAG-3′) were used with a program consisting of an initial denaturation and *Taq* polymerase activation step of 10 s at 95 °C, followed by 40 cycles of 10 s at 95 °C and 31 s at 60 °C. Primer specificity was confirmed by amplicon dissociation curves. The absence of genomic DNA was confirmed by PCR on RNA samples without reverse transcription. Expression levels were normalized to that of *LjeIF-4A* used as an internal reference gene. Sequences of all genes used in this study were retrieved from the *Lotus japonicus* Genome Sequencing Project (http://www.kazusa.or.jp/lotus/).

### 2.9. Histochemical Detection of H_2_O_2_ and O_2_^−^

H_2_O_2_ was detected in situ according to Thordal-Christensen et al. [46] and Signorelli et al. [47]. Detached leaves and roots were vacuum-infiltrated in the dark with 10 mM potassium phosphate buffer, 10 mM NaN_3_, and 0.1% (w/v) 3,3′-diaminobenzidine (DAB), pH 7.8. Samples were incubated overnight in the dark, then cleared with 0.15% (w/v) trichloroacetic acid in 4:1 (v/v) ethanol: chloroform for 48 h, and photographed.

Superoxide radical (O_2_^−^) was detection in situ essentially as described by Jabs et al. [48] and Signorelli et al. [47]. Detached leaves and roots were vacuum-infiltrated with 10 mM potassium phosphate buffer, 10 mM NaN_3_, 0.1% (w/v) nitro blue tetrazolium (NBT), and 0.05% (v/v) Tween 20, pH 7.8. Treated samples were then maintained for 30 min under daylight, cleared as above, and photographed.

### 2.10. Light Microscopy

Nodules were fixed with 4% paraformaldehyde and 2.5% glutaraldehyde in 0.1 M sodium phosphate buffer (pH 7.2) at 4 °C overnight. The fixed samples were dehydrated through a graded ethanol series, embedded in JB4 resin (Polysciences Inc., Warrington, PA, USA), and sectioned (3 μm thick). Sections were stained with the Periodic Acid-Schiff (PAS) reagent (Muto Pure Chemicals, Tokyo, Japan) according to the manufacturer’s instructions.

## 3. Results

### 3.1. Nodules of Ox1 and Afx1 Lines have High Nitrogenase Activity and Low NO Levels

**Figure 1:**
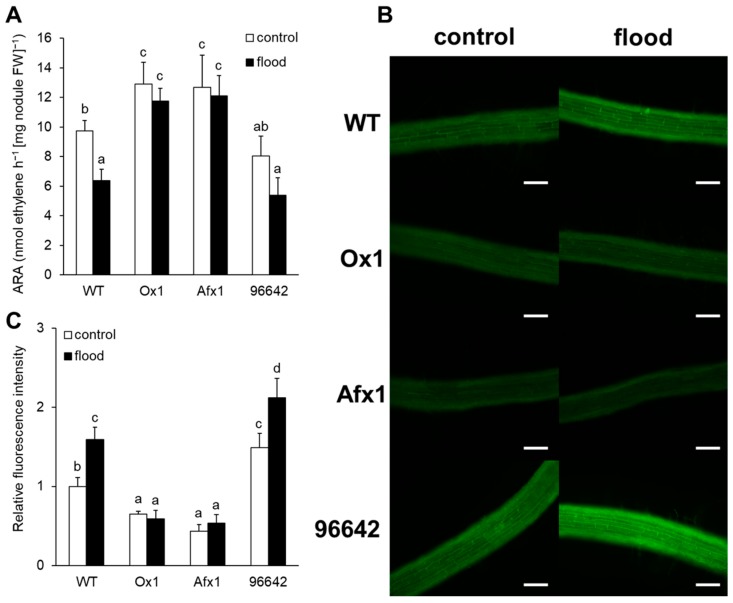
Nitrogenase activity and NO production in roots of the WT, Ox1, Afx1, and 96642 lines. (**A**) Nitrogenase activity (estimated as ARA) was measured in the flooded and unflooded (control) nodules and was expressed as ethylene produced per hour and mg nodule fresh weight. (**B**) Fluorescence imaging of NO production in roots with the DAF-FM DA probe. Scale bars, 100 µm. (**C**) Quantification of fluorescence intensity in DAF-FM DA images. In A and C, values are means ± SE of nine biological replicates. Means denoted by the same letter do not differ significantly by Student’s *t*-test at *p* < 0.05.

Without flooding, the ARA level was significantly higher in Ox1 and Afx1 than in WT and 96642 plants (Figure 1A). Flooding significantly reduced the ARA level in WT; it tended to reduce it in 96642 but had no effect in Ox1 and Afx1 (Figure 1A).

Using the cell-permeable DAF-FM DA NO probe, we compared the endogenous production of NO in the roots of WT and transgenic plants under flooded and control conditions. In line with our previous findings [27,33], endogenous NO levels were lower in the roots of Ox1 and Afx1 and higher in the roots of 96642 than in WT (Figure 1B,C). Flooding increased endogenous NO levels in WT and 96642 but not in Ox1 or Afx1 (Figure 1B,C).

**Figure 2:**
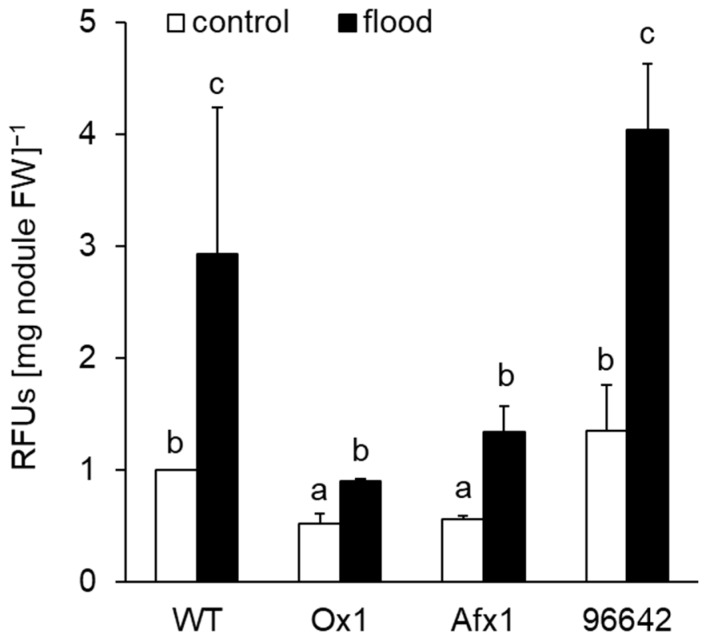
Quantification of NO released from nodules with the DAF-FM probe. Values are means ± SE of nine biological replicates. Means denoted by the same letter do not differ significantly by Student’s *t*-test (*p* < 0.05).

To assess the NO levels released by nodules, we used the non-cell-permeable DAF-FM probe. Flooding significantly increased NO release from the nodules of all lines tested, although the levels remained significantly lower in Ox1 and Afx1 than in WT and 96642 (Figure 2).

### 3.2. Glb1s Alleviate Nodule Senescence Caused by Flooding

**Figure 3:**
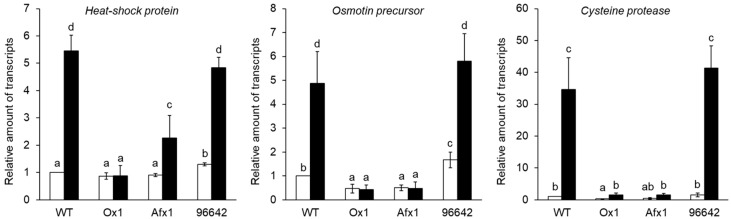
Expression of senescence-associated genes in flooded and unflooded (control) nodules. For each line, the mRNA levels in unflooded WT nodules were set at 1. Values are means ± SE of three biological replicates, each with three technical replicates. Open bars, unflooded; filled bars, flooded. Means denoted by the same letter do not differ significantly by Student’s *t*-test at *p* < 0.05.

We have reported that nodule senescence in the Ox1 line is delayed [33]. Quantification by qRT-PCR showed that the expression of all three nodule senescence marker genes (coding a heat shock protein, an osmotin precursor, and cysteine protease Cyp2; [33,49,50]) was increased in flooded nodules relative to untreated nodules of WT and 96642; the increase was much lower or absent in the nodules of Ox1 and Afx1 (Figure 3).

**Figure 4:**
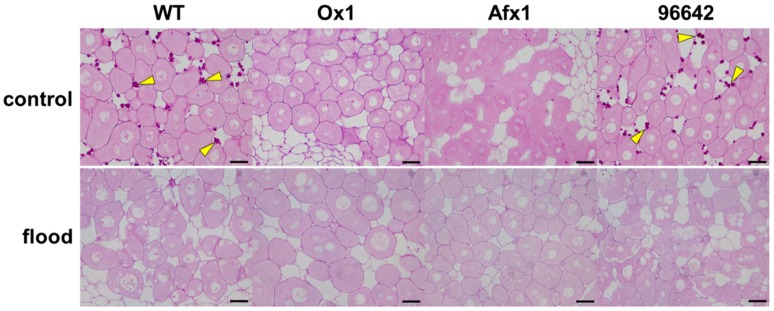
Microscopic images of nodules of WT, Ox1, Afx1, and 96642 plants. Sections of flooded or unflooded (control) nodules were stained with PAS to visualize infected cells and starch granules (arrowheads). Scale bars, 20 µm.

Microscopic examination of nodule sections stained with PAS reagent revealed that flooding increased the number and size of vacuoles in the infected cells of WT and 96642, indicating nodule senescence [51], but not in those of Ox1 and Afx1 lines (Figure 4). However, accumulation of starch granules—another typical phenotype of nodule senescence—was not observed in the nodules of WT and 96642 (Figure 4).

### 3.3. Glb1s Alleviate the Effects of Flooding in Leaves and Roots

**Figure 5:**
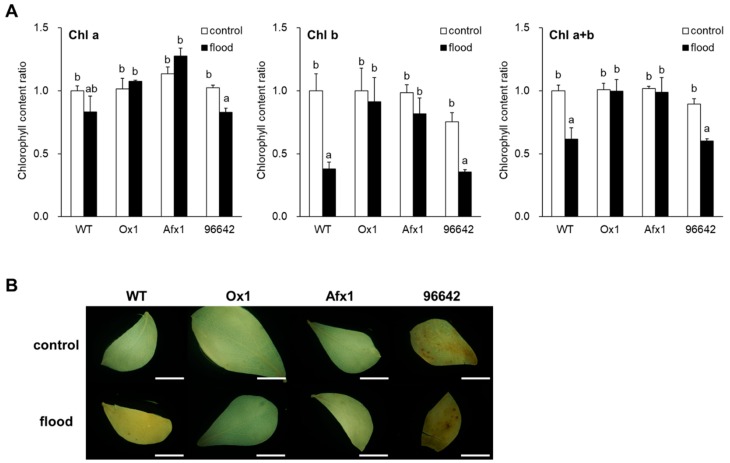
Relative chlorophyll content and ROS detection in flooded and unflooded (control) plants. (**A**) Absorbance of chlorophylls (*a*, *b*, *a*+*b*) dissolved in DMF. Chlorophyll content in the leaves of unflooded WT was set at 1. Values are means ± SE of three biological replicates, each with three technical replicates. Means denoted by the same letter do not differ significantly by Student’s *t*-test (*p* < 0.05). (**B**) In situ hydrogen peroxide staining with DAB in leaves of flooded and unflooded plants. Scale bars, 5 mm.

**Figure 6:**
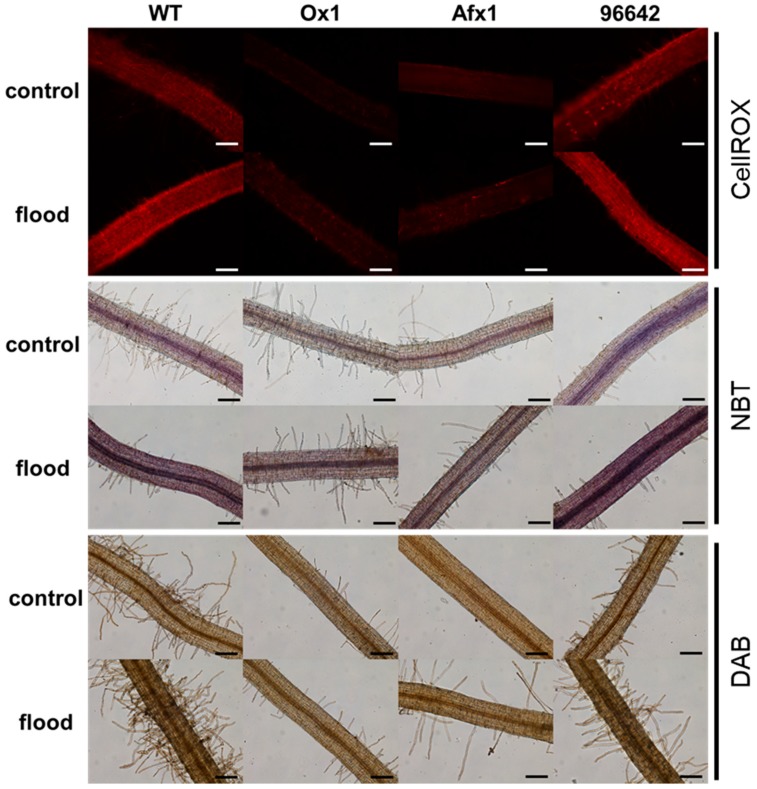
ROS in roots of flooded and unflooded (control) plants. Oxidative activity of ROS was detected with the CellROX probe, superoxide with NBT and hydrogen peroxide with DAB. Scale bars, 100 µm.

Flooding reduced total leaf chlorophyll contents in WT and 96642, mostly because of the decrease in chlorophyll-*b*, but not in Ox1 and Afx1 (Figure 5A). Flooding increased the intensity of DAB staining (indicator of H_2_O_2_ level) of WT and 96642 leaves, but no obvious effect was observed in Ox1 and Afx1 (Figure 5B). Unexpectedly, NBT (indicator of O_2_^−^ level) strongly stained leaves of all lines without flooding, whereas flooding decreased leaf staining in all lines, with no obvious differences among them (Supplementary Figure S1). Electrolyte leakage rate did not show significant differences among the lines or treatments (Supplementary Figure S2). In roots, flooding increased the fluorescence intensity of CellROX reagent in WT and 96642, but it remained low in Ox1 and Afx1 (Figure 6, Supplementary Figure S3). Although NBT and DAB staining was weaker in the roots of Ox1 and Afx1 than in those of WT and 96642 regardless of flooding, the differences among the lines were less clear with DAB staining than with CellROX staining (Figure 6).

## 4. Discussion

Flooding inhibits plant growth by causing hypoxic stress in roots [52], which induces NO production and Glb1 expression [6,12,16]. Overexpression of Glb1 enhances NO scavenging activity, increases tolerance to hypoxic stress [6,16,53], increases nitrogenase activity, and slows down the aging of nodule symbiosis [33].

In this study, the lines of *L. japonicus* stably transformed with *LjGlb1-1* (Ox1) or *AfGlb1* (Afx1) allowed us to study the effect of Glb1 overexpression on the hypoxic tolerance of nodule symbiosis. Flooding significantly decreased nitrogenase activity and increased the NO level of WT and 96642 nodules but did not alter the high nitrogenase activity and low NO level in the Ox1 and Afx1 nodules (Figure 1 and Figure 2). These results suggest that increased Glb1 expression enhanced NO-scavenging activity and improved flooding tolerance of the nodules. Flooding increased the expression of three senescence-related genes more in WT and 96642 than in Ox1 and Afx1 (Figure 3); the number and size of the vacuoles of the infected nodule cells were increased in WT and 96642, which is typical of aged nodules [51]. The accumulation of starch granules in the nodule also indicates senescence. The decreased nitrogenase activity of the aged nodules does not consume the product of photosynthesis transported from the leaves, and the residual carbon source accumulates as starch granules. However, starch granules were not observed in any lines despite the decrease in nitrogenase activity in WT and 96642 (Figure 4). Flooding significantly reduces the photosynthetic capacity and transpiration rate [2,54]; in our experiments, flooding might have reduced the photosynthetic capacity or the transport of photosynthetic products to roots.

Soil flooding can perturb the photosynthetic machinery, reducing photosynthetic potential, possibly because of ROS production [1]. ROS damage the structure of chloroplast membranes and inhibit the photosystem function [2]. In the present study, flooding decreased the total amount of chlorophylls in WT and 96642, but not in Ox1 or Afx1 (Figure 5A). Leaf DAB staining was strong in 96642 and flooding increased it in WT, with no obvious difference in Ox1 and Afx1. These results suggest that flooding increased H_2_O_2_ levels in leaves, and that Glb1s were involved. Flooding reduced the leaf O_2_^−^ levels in all lines, with no obvious difference among them (Supplementary Figure S1). ROS lead to electrolyte leakage [3,4,5], but we detected no significant difference among the lines in electrolyte leakage rate (Supplementary Figure S2), despite the higher level of H_2_O_2_ in the leaves of WT and 96642 (Figure 5B). The metabolism might have adapted to flooding to reduce the damage by ROS.

During flooding, roots are exposed to hypoxic stress more than the other plant parts. In our study, ROS levels increased in the roots and leaves of WT and 96642 (Figure 5B and Figure 6), but ROS and NO levels remained low in the lines with increased Glb1 levels (Figure 1B,C and Figure 6). Although the results of NBT and DAB were similar to those of CellROX fluorescence in roots, the difference among the lines in DAB staining was less clear than that in CellROX staining (Figure 6), possibly because of different sensitivity and specificity of these staining methods: CellROX detects various ROS including H_2_O_2_ and O_2_^−^, whereas DAB detects only H_2_O_2_, and NBT detects only O_2_^−^. Under control conditions, there was no difference in the amount of ROS between the roots of 96642 and WT, but it was slightly higher in the leaves of 96642. We do not have a good explanation for these inconsistent results. The null mutant 96642 showed higher NO levels in the roots (Figure 1B,C) and higher expression levels of some senescence-related genes in the nodules than WT (Figure 3). These physiological differences might affect the amount of ROS in leaves. In cultured alfalfa roots, Glb1 overexpression improves the antioxidant status by increasing ascorbate levels and the activity of enzymes involved in H_2_O_2_ metabolism [55]. In corn plants, Glb1 overexpression alleviates flooding stress by limiting ROS-induced damage and ensuring a sustained photosynthetic rate through improvement of the ascorbate antioxidant status and an increase in activities of several ROS-scavenging enzymes [56]. In *L. japonicus*, Glb1 overexpression might alleviate flooding stress by improving ROS metabolism, although we did not investigate the expression of genes related to the regulation of ROS.

## 5. Conclusions

Glb1 contributes to maintaining nodule symbiosis under flooding conditions and controls ROS by scavenging NO.

## Supplementary Materials

The following are available online at https://www.mdpi.com/2076-3921/8/7/206/s1. Figure S1. In situ superoxide staining with NBT. Scale bars, 5 mm. Figure S2. Electrolyte leakage rate during flooding of detached leaves. Figure S3. Effect of flooding on the relative amount of ROS in roots.

## References

[1] Chen Y., Chen X., Wang H., Bao Y., Zhang W. (2014). Examination of the leaf proteome during flooding stress and the induction of programmed cell death in maize. Proteome Sci..

[2] Titarenko T. (2000). Test parameters of revealing the degree of fruit plants tolerance to the root hypoxia caused by flooding of soil. Plant Physiol. Biochem..

[3] Hetherington A.M., Hunter S., Crawford R.M.M. (1982). Contrasting effects of anoxia on rhizome lipids in Iris species. Phytochemistry.

[4] Crawford R.M.M., Walton J.C., Wollenweber-Ratzer W. (1994). Similarities between post-ischaemic injury to animal tissues and post-anoxic injury in plants. Proc. R. Soc. Edinb..

[5] Jambunathan N. (2010). Determination and detection of reactive oxygen species (ROS), lipid peroxidation, and electrolyte leakage in plants. Methods Mol. Biol..

[6] Dordas C., Hasinoff B.B., Igamberdiev A.U., Manac’h N., Rivoal J., Hill R.D. (2003). Expression of a stress-induced hemoglobin affects NO levels produced by alfalfa root cultures under hypoxic stress. Plant J..

[7] Dordas C., Rivoal J., Hill R.D. (2003). Plant haemoglobins, nitric oxide and hypoxic stress. Ann. Bot..

[8] Dordas C., Hasinoff B.B., Rivoal J., Hill R.D. (2004). Class-1 hemoglobins, nitrate and NO levels in anoxic maize cell-suspension cultures. Planta.

[9] Fukao T., Serres J.B. (2004). Plant responses to hypoxia-is survival a balancing act?. Trends Plant Sci..

[10] García-Mata C., Lamattina L. (2002). Nitric oxide and abscisic acid cross talk in guard cells. Plant Physiol..

[11] Joudoi T., Shichiri Y., Kamizono N., Akaike T., Sawa T., Yoshitake J., Yamada N., Iwai S. (2013). Nitrated cyclic gmp modulates guard cell signaling in *Arabidopsis*. Plant Cell.

[12] Igamberdiev A.U., Hill R.D. (2004). Nitrate, NO and haemoglobin in plant adaptation to hypoxia: An alternative to classic fermentation pathways. J. Exp. Bot..

[13] Hebelstrup K.H., Shah J.K., Igamberdiev A.U. (2013). The role of nitric oxide and hemoglobin in plant development and morphogenesis. Physiol. Plant..

[14] Trevaskis B., Watts R.A., Andersson C.R., Llewellyn D.J., Hargrove M.S., Olson J.S., Dennis E.S., Peacock W.J. (1997). Two hemoglobin genes in *Arabidopsis thaliana*: The evolutionary origins of leghemoglobins. Proc. Natl. Acad. Sci. USA.

[15] Watts R.A., Hunt P.W., Hvitved N.A., Hargrove M.S., Peacock W.J., Dennis E.S. (2001). A hemoglobin from plants homologous to truncated hemoglobins of microorganisms. Proc. Natl. Acad. Sci. USA.

[16] Hunt P.W., Klok E.J., Trevaskis B., Watts R.A., Ellis M.H., Peacock W.J., Dennis E.S. (2002). Increased level of hemoglobin 1 enhances survival of hypoxic stress and promotes early growth in *Arabidopsis thaliana*. Proc. Natl. Acad. Sci. USA.

[17] Smagghe B.J., Hoy J.A., Percifield R., Kundu S., Hargrove M.S., Sarath G., Hilbert J.L., Watts R.A., Dennis E.S., Peacock W.J. (2009). Correlations between oxygen affinity and sequence classifications of plant hemoglobins. Biopolymers.

[18] Kubo H. (1939). Über hämoprotein aus den wurzelknöllchen von leguminosen. Acta Phytochim..

[19] Ott T., van Dongen J.T., Günther C., Krusell L., Desbrosses G., Vigeolas H., Bock V., Czechowski T., Geigenberger P., Udvardi M.K. (2005). Symbiotic leghemoglobins are crucial for nitrogen fixation in legume root nodules but not for general plant growth and development. Curr. Biol..

[20] Vieweg M.F., Hohnjec N., Küster H. (2005). Two genes encoding different truncated hemoglobins are regulated during root nodule and arbuscular mycorrhiza symbioses of *Medicago truncatula*. Planta.

[21] Sáenz-Rivera J., Sarath G., Arredondo-Peter R. (2004). Modeling the tertiary structure of a maize (Zea mays ssp. mays) non-symbiotic hemoglobin. Plant Physiol. Biochem..

[22] Perazzolli M., Dominici P., Romero-Puertas M.C., Zago E., Zeier J., Sonoda M., Lamb C., Delledonne M. (2004). *Arabidopsis* nonsymbiotic hemoglobin AHb1 modulates nitric oxide bioactivity. Plant Cell.

[23] Lee B.R., Hwang S. (2015). Over-expression of *NtHb1* encoding a non-symbiotic class 1 hemoglobin of tobacco enhances a tolerance to cadmium by decreasing NO (nitric oxide) and Cd levels in *Nicotiana tabacum*. Environ. Exp. Bot..

[24] Bahmani R., Kim D., Na J., Hwang S. (2019). Expression of the tobacco non-symbiotic class 1 hemoglobin gene Hb1 reduces cadmium levels by modulating Cd transporter expression through decreasing nitric oxide and ROS level in *Arabidopsis*. Front. Plant Sci..

[25] Uchiumi T., Shimoda Y., Tsuruta T., Mukoyoshi Y., Suzuki A., Senoo K., Sato S., Kato T., Tabata S., Higashi S. (2002). Expression of symbiotic and nonsymbiotic globin genes responding to microsymbionts on *Lotus japonicus*. Plant Cell Physiol..

[26] Bustos-Sanmamed P., Tovar-Méndez A., Crespi M., Sato S., Tabata S., Becana M. (2011). Regulation of nonsymbiotic and truncated hemoglobin genes of *Lotus japonicus* in plant organs and in response to nitric oxide and hormones. New Phytol..

[27] Fukudome M., Calvo-Begueria L., Kado T., Osuki K., Rubio M.C., Murakami E., Nagata M., Kucho K., Sandal N., Stougaard J. (2016). Hemoglobin LjGlb1-1 is involved in nodulation and regulates the level of nitric oxide in the *Lotus japonicus*-*Mesorhizobium loti* symbiosis. J. Exp. Bot..

[28] Nagata M., Murakami E., Shimoda Y., Shimoda-Sasakura F., Kucho K., Suzuki A., Abe M., Higashi S., Uchiumi T. (2008). Expression of a class 1 hemoglobin gene and production of nitric oxide in response to symbiotic and pathogenic bacteria in *Lotus japonicus*. Mol. Plant-Microbe Interact..

[29] Trinchant J.C., Rigaud J. (1982). Nitrite and nitric oxide as inhibitors of nitrogenase from soybean bacteroids. Appl. Environ. Microbiol..

[30] Kato K., Kanahama K., Kanayama Y. (2010). Involvement of nitric oxide in the inhibition of nitrogenase activity by nitrate in Lotus root nodules. J. Plant Physiol..

[31] Cam Y., Pierre O., Boncompagni E., Hérouart D., Meilhoc E., Bruand C. (2012). Nitric oxide (NO): A key player in the senescence of *Medicago truncatula* root nodules. New Phytol..

[32] Shimoda Y., Shimoda-Sasakura F., Kucho K., Kanamori N., Nagata M., Suzuki A., Abe M., Higashi S., Uchiumi T. (2009). Overexpression of class 1 plant hemoglobin genes enhances symbiotic nitrogen fixation activity between *Mesorhizobium loti* and *Lotus japonicus*. Plant J..

[33] Fukudome M., Watanabe E., Osuki K., Imaizumi R., Aoki T., Becana M., Uchiumi T. (2019). Stably-transformed *Lotus japonicus* plants overexpressing phytoglobin *Ljglb1-1* show decreased nitric oxide levels in roots and nodules as well as delayed nodule senescence. Plant Cell Physiol..

[34] Sasakura F., Uchiumi T., Shimoda Y., Suzuki A., Takenouchi K., Higashi S., Abe M. (2006). A class 1 hemoglobin gene from *Alnus firma* functions in symbiotic and nonsymbiotic tissues to detoxify nitric oxide. Mol. Plant-Microbe Interact..

[35] Schwintzer C.R. (1985). Effect of spring flooding on endophyte differentiation, nitrogenase activity, root growth and shoot growth in *Myrica gale*. Plant Soil..

[36] Shimamura S., Mochizuki T., Nada Y., Fukuyama M. (2002). Secondary paerenchyma formation and its relation to nitrogen fixation in root nodules of soybean plants (*Glycine max*) grown under flooded conditions. Plant Prod. Sci..

[37] Sánchez C., Gates A.J., Meakin G.E., Uchiumi T., Girard L., Richardson D.J., Bedmar E.J., Delgado M.J. (2010). Production of nitric oxide and nitrosylleghemoglobin complexes in soybean nodules in response to flooding. Mol. Plant-Microbe Interact..

[38] Fukai E., Soyano T., Umehara Y., Nakayama S., Hirakawa H., Tabata S., Sato S., Hayashi M. (2012). Establishment of a *Lotus japonicus* gene tagging population using the exon-targeting endogenous retrotransposon LORE1. Plant J..

[39] Urbański D.F., Małolepszy A., Stougaard J., Andersen S.U. (2012). Genome-wide LORE1 retrotransposon mutagenesis and high-throughput insertion detection in *Lotus japonicus*. Plant J..

[40] Małolepszy A., Mun T., Sandal N., Gupta V., Dubin M., Urbański D., Shan N., Bachmann A., Fukai E., Hirakawa H. (2016). The *LORE1* insertion mutant resource. Plant J..

[41] Aoki T., Kamizawa A., Ayabe S. (2002). Efficient *Agrobacterium*-mediated transformation of *Lotus japonicus* with reliable antibiotic selection. Plant Cell Rep..

[42] Kaneko T., Nakamura Y., Sato S., Asamizu E., Kato T., Sasamoto S., Watanabe A., Idesawa K., Ishikawa A., Kawashima K. (2000). Complete genome structure of the nitrogen-fixing symbiotic bacterium *Mesorhizobium loti*. DNA Res..

[43] Fåhraues G. (1957). The infection of clover root hair by nodule bacteria studied by a single glass slide technique. Microbiology.

[44] Porra R.J., Thompson W.A., Kriedemann P.E. (1989). Determination of accurate extinction coefficients and simultaneous equations for assaying chlorophylls a and b extracted with four different solvents: Verification of the concentration of chlorophyll standards by atomic absorption spectroscopy. Biochim. Biophys. Acta Bioenerg..

[45] Rolny N., Costa L., Carrión C., Guiamet J.J. (2011). Is the electrolyte leakage assay an unequivocal test of membrane deterioration during leaf senescence?. Plant Physiol. Biochem..

[46] Thordal-Christensen H., Zhang Z., Wei Y., Collinge D.B. (1997). Subcellular localization of H_2_O_2_ in plants. H_2_O_2_ accumulation in papillae and hypersensitive response during the barley-powdery mildew interaction. Plant J..

[47] Signorelli S., Corpas F.J., Borsani O., Barroso J.B., Monza J. (2013). Water stress induces a differential and spatially distributed nitro-oxidative stress response in roots and leaves of *Lotus japonicus*. Plant Sci..

[48] Jabs T., Dietrich R.A., Dangl J.L. (1996). Initiation of runaway cell death in an *Arabidopsis* mutant by extracellular superoxide. Science.

[49] Fujie M., Shintaku H., Maeno H., Kajihara R., Usami S., Yamada T. (2009). Molecular cytological analysis of cysteine proteinases from nodules of *Lotus japonicus*. Cytologia.

[50] Chungopast S., Hirakawa H., Sato S., Hanada Y., Saito K., Kawaguchi M., Tajima S., Nomura M. (2014). Transcriptomic profiles of nodule senescence in *Lotus japonicus* and *Mesorhizobium loti* symbiosis. Plant Biotechnol..

[51] Hossain M.S., Umehara Y., Kouchi H. (2006). A novel fix-symbiotic mutant of *Lotus japonicus*, Ljsym105, shows impaired development and premature deterioration of nodule infected cells and symbiosomes. Mol. Plant Microbe Interact..

[52] Rocha M., Licausi F., Araújo W.L., Nunes-Nesi A., Sodek L., Fernie A.R., Van Dongen J.T. (2010). Glycolysis and the tricarboxylic acid cycle are linked by alanine aminotransferase during hypoxia induced by waterlogging of *Lotus japonicus*. Plant Physiol..

[53] Mira M., Hill R.D., Stasolla C. (2016). Regulation of programmed cell death by phytoglobins. J. Exp. Bot..

[54] Jackson M.B. (2002). Long-distance signalling from roots to shoots assessed: The flooding story. J. Exp. Bot..

[55] Igamberdiev A.U., Stoimenova M., Seregélyes C., Hill R.D. (2006). Class-1 hemoglobin and antioxidant metabolism in alfalfa roots. Planta.

[56] Youssef M.S., Mira M.M., Renault S., Hill R.D., Stasolla C. (2016). Phytoglobin expression influences soil flooding response of corn plants. Ann. Bot..

