# Supplementary material for: Ectopic or Over-Expression of Class 1 Phytoglobin Genes Confers Flooding Tolerance to the Root Nodules of Lotus japonicus by Scavenging Nitric Oxide
Source: Antioxidants (Basel). 2019 Jul 4;8(7):206. doi: 10.3390/antiox8070206 (PMC6681080; doi:10.3390/antiox8070206)
Supplement: Supplementary file 1 [file antioxidants-08-00206-s001.pdf]

antioxidants

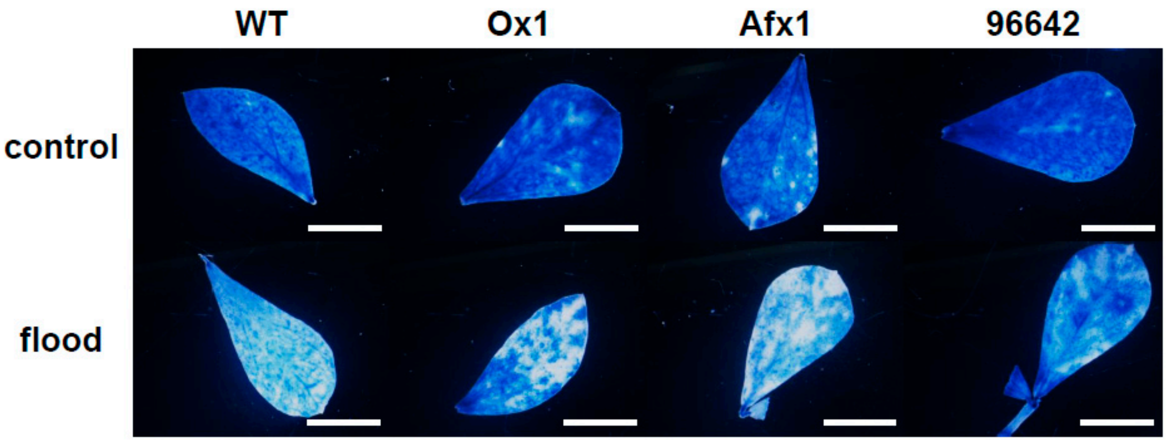

Figure S1. In situ superoxide staining with NBT. Scale bars, 5 mm.

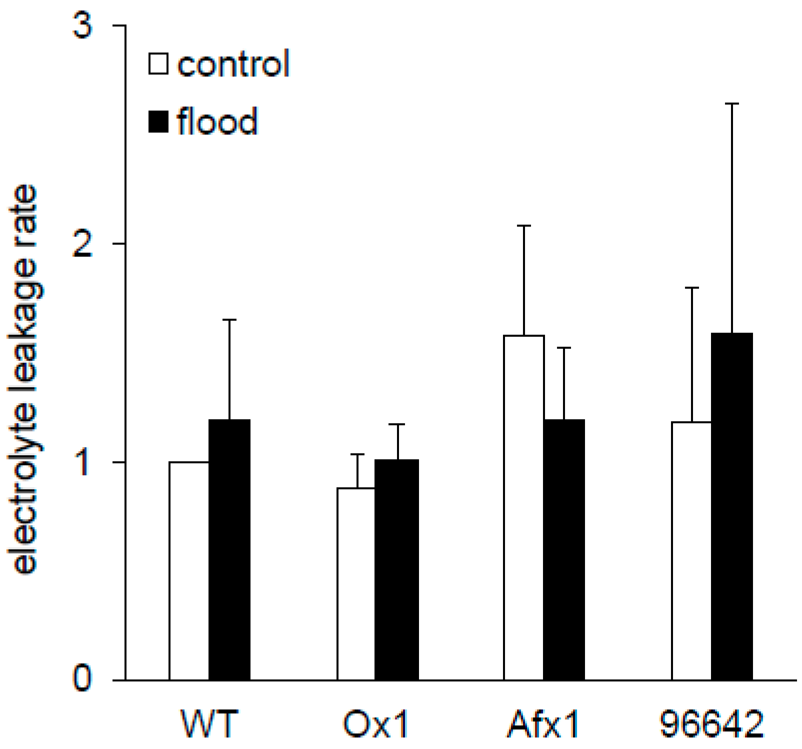

Figure S2. Electrolyte leakage rate during flooding of detached leaves. Bars indicate the ratio to the control WT. No significant differences were detected by Student's *t*-test ($p < 0.05$).

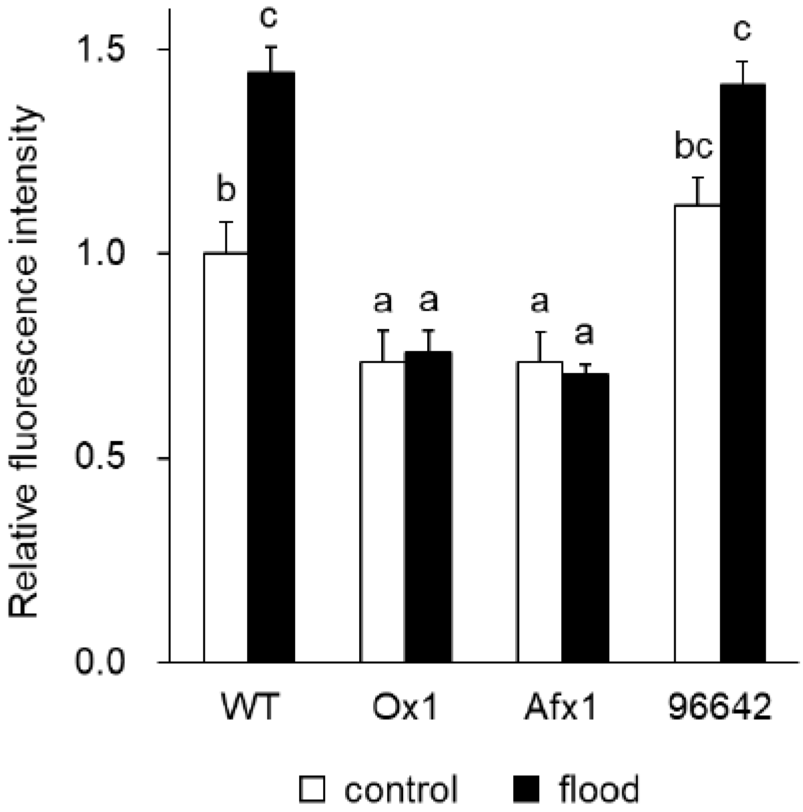

Figure S3. Effect of flooding on the relative amount of ROS in roots. CellROX fluorescence images (Figure 6) were quantified with the image analysis software ImageJ. Values are means ± SE of nine biological replicates. Means denoted by the same letter do not differ significantly by Student's *t*-test ($p < 0.05$).
